# Individual and Institutional Factors Associated With Urinary Incontinence Among Nursing Home Residents: A Multilevel Analysis

**DOI:** 10.1111/jan.16986

**Published:** 2025-04-16

**Authors:** Javier Jerez‐Roig, Nayara Priscila Dantas de Oliveira, Pau Moreno‐Martin, Júlia Casacuberta‐Roca, Dyego Leandro Bezerra de Souza, Ester Goutan‐Roura, Laura Coll‐Planas, Adrian Wagg, William Gibson, Xavier Gómez‐Batiste

**Affiliations:** ^1^ Research Group on Methodology, Methods, Models and Outcomes of Health and Social Sciences (M_3_O), Faculty of Health Sciences and Welfare, Centre for Health and Social Care Research (CESS) University of Vic—Central University of Catalonia (UVic‐UCC) Vic Spain; ^2^ Institute for Research and Innovation in Life Sciences and Health in Central Catalonia (IRIS‐CC) Vic Spain; ^3^ Department of Health Promotion and Rehabilitation, Institute of Sport Science and Innovations Lithuanian Sports University Kaunas Lithuania; ^4^ Graduate Program in Rehabilitation and Functional Performance Physiotherapy Department, University of Pernambuco Petrolina Brazil; ^5^ Graduate Program in Collective Health, Department of Collective Health Federal University of Rio Grande do Norte Natal Brazil; ^6^ Division of Geriatric Medicine, Department of Medicine University of Alberta Edmonton Alberta Canada; ^7^ Chair of Palliative Care, Central Catalonia Chronicity Research Group (C3RG), Centre for Health and Social Care Research (CESS), Faculty of Medicine University of Vic—Central University of Catalonia Vic Spain

**Keywords:** associated factors, multilevel analysis, nursing homes, older adults, urinary incontinence

## Abstract

**Aims:**

(1) To analyse individual and institutional‐level factors associated with urinary incontinence in older adults living in nursing homes; (2) to estimate the prevalence of urinary, faecal and double incontinence in nursing home residents.

**Design:**

Cross‐sectional study.

**Methods:**

Residents aged 65+ living in 22 nursing homes in Catalonia (Spain) were included. Descriptive, bivariate, and multilevel analyses were performed.

**Results:**

The final sample comprised 452 residents (75.9% female, mean age of 87.0 years). The prevalence of urinary, faecal and double incontinence was 77.5%, 46.1% and 45.7%, respectively. Urinary incontinence was statistically significantly associated with neurological conditions, moderate cognitive impairment, moderate dementia, severe cognitive impairment, very severe cognitive impairment and age.

**Conclusion:**

Approximately three out of four nursing home residents suffered from urinary incontinence and almost half of the sample from faecal or double incontinence. Individual‐level factors (cognition, neurological conditions and age) played a more important role than institutional‐level factors for urinary incontinence.

**Implications for the Profession and Patient Care:**

The findings of this study highlight the importance of individual‐level interventions to prevent and manage urinary incontinence in nursing homes.

**Impact:**

In Catalonian nursing homes, individual factors such as cognitive impairment and neurological conditions were more strongly associated with urinary incontinence than institutional factors. This has implications for improving care provided to older adults, particularly those with dementia and neurological conditions.

**Reporting Method:**

STROBE (Strengthening the Reporting of Observational Studies in Epidemiology) guidelines.

**Patient or Public Contribution:**

Nursing home residents were not involved in this study.

**Summary**

- What does this paper contribute to the wider global clinical community?
  ○ Knowledge on the prevalence of urinary, faecal and double incontinence in nursing home residents, as well as factors associated with urinary incontinence at both the individual and institutional levels, which allows planning and evaluation of interventions designed to ameliorate the problem.

## 1 Introduction

Bladder health is essential in the maintenance of independence and functional performance (World Health Organization 2015; European Association of Urology 2023). Urinary incontinence (UI) is a common condition with a profound impact on bladder health, quality of life (physical, emotional, and social impact) and well‐being (World Health Organization 2015). UI, recognised as a geriatric syndrome, defined as the involuntary loss of urine (Abrams et al. 2002), affects up to 55–60 million people in Europe (European Association of Urology 2023). In Europe, the annual economic burden of UI was estimated as €69 billion (2023), equivalent to half the direct medical cost of diabetes and at least two thirds of the economic burden of cancer (European Association of Urology 2023). These costs will increase with the projected 25% increase in UI prevalence to almost €87 billion in 2030 (€100 billion including caregiver costs) if no action is taken (European Association of Urology 2023; Milsom et al. 2014).

Incontinence is especially prevalent in nursing homes (NHs), where UI affects more than 60%–75% of residents (Jerez‐Roig et al. 2024; Onder et al. 2012). These older adults comprise the frailest, most vulnerable sector of society, often towards the end of life (Onder et al. 2012; Escribà‐Salvans et al. 2024). UI in frail older adults is associated with multiple factors, which can be classified in environmental (institutional) and individual (including comorbidity or cognitive and functional impairments) (Gibson et al. 2021). Institutional factors are those related to the physical environment (e.g., built architecture) and organisational processes of care (Cardozo et al. 2023). Physical environmental factors may contribute to UI in older adults, but there is still limited research on this topic (Cardozo et al. 2023). Organisational factors include aspects such as staff‐to‐resident ratios, care protocols, provision of continence care, staff knowledge on UI or and attitudes to incontinence as a health condition (Gibson et al. 2021). Common issues in processes of care include lack of knowledge and assessment of UI among NH staff, practices that restrict mobility and functional ability of residents, difficulties in access to toileting assistance, reduced staffing levels and overuse of absorbent products (Cardozo et al. 2023).

Continence care in NHs often focuses on containment rather than assessment and treatment. The International Consultation on Incontinence emphasises the need for a shift towards evidence‐based management strategies. For instance, behavioural interventions, such as prompted voiding or bladder training, have been shown to significantly reduce UI episodes when consistently applied by a multiprofessional team (Cardozo et al. 2023).

Institutional factors, such as the availability of well‐trained caregivers and appropriate toileting facilities, can affect residents' continence status (Namazi et al. 1991). Creating an environment that supports continence, such as ensuring accessible toilets, adequate lighting, and clear signage, can help reduce incontinence among older adults with dementia (Namazi et al. 1991). In NH, cognitive impairment often leads to reduced awareness of bladder control, making timely toileting assistance a crucial intervention by care staff (Murphy et al. 2021).

Nurses are central to UI management and care planning, as they are involved in the assessment of bladder function, implementation of individualised toileting schedules and providing education on continence care strategies for both residents and caregivers (Wagg et al. 2023).

## 2 Background

The prevalence of UI in NHs is high, mainly due to factors such as cognitive impairment, mobility limitations and chronic medical conditions, making nurse‐led strategies essential for effective management and prevention (Gibson et al. 2021; Bliss et al. 2018). Nurses are responsible for assessing residents' continence status, and along with nursing assistants (also known as care aides, personal care workers), implementing and monitoring programs to improve bladder health (Wagg et al. 2023; Offermans et al. 2009). Behavioural interventions, including toileting programs, fluid training, and (if possible) pelvic floor muscle training, can be effective in managing UI and improving residents' quality of life (Marcu et al. 2024). This is in addition to reviewing medications that contribute to UI, ensuring their appropriate prescription and optimising the management of comorbid conditions which might impair the ability to successfully toilet (British Geriatrics Society, n.d.).

Organisational factors, including institutional policies, staffing models, care processes, access to continence products, and collaboration with multidisciplinary teams, significantly affect the success of nursing interventions in UI management (Yoon et al. 2012; Lyons 2010). Adequate staffing levels and proper training in UI management are critical, as understaffed facilities may struggle to provide timely toileting assistance, increasing the risk of incontinence‐related complications such as incontinence associated dermatitis, skin breakdown and infections (Cardozo et al. 2023; Blekken et al. 2016).

A scoping review on interventions for the management of UI in NHs recommended a multicomponent approach led by a specialist like a nurse with advanced clinical expertise and leadership abilities (Cambridge Media Journals n.d.). Environmental attributes, including team cohesiveness, consistent assignment, and staff cohesion, can affect residents' urinary continence status (Cambridge Media Journals n.d.; Perruchoud et al. 2021). NH policies and practices should prioritise staff education programs and implement interventions that enhance residents' autonomy, engagement in activities, nutritional well‐being, hydration and toileting (Cambridge Media Journals n.d.). A systematic review found that education improves nurses' and nursing assistants' knowledge and attitudes towards UI, which might lead to better continence care practices and improved patient outcomes (Ostaszkiewicz et al. 2020).

Multilevel analysis can help understand the influence of these factors on UI, by providing estimates of any institutional effect, isolated from the residents and accounting for random effects for each institution (Serrano‐Urrea et al. 2017). Previous studies have used this type of analysis to understand these factors in conditions such as functional disability (Serrano‐Urrea et al. 2017), falls (do Nascimento et al. 2018) or frailty (Xu et al. 2022). However, to the best of our knowledge, no studies have used multilevel analysis to assess individual and institutional factors operating in NH residents. Furthermore, there is no published comprehensive information on the continence status of a representative sample of NH residents in Catalonia (Spain).

## 3 The Study

### 3.1 Aims

The main objective of this study was to examine the impact of individual and institutional factors associated with UI among older adults living in NHs in Catalonia. A secondary objective was to estimate the prevalence of UI, faecal incontinence (FI) and double (urinary and faecal) incontinence.

### 3.2 Study Design, Setting and Participants

This was a multicentre cross‐sectional study reported according to STROBE (Strengthening the Reporting of Observational Studies in Epidemiology) guidelines (von Elm et al. 2007). It was a subset of a larger project (ResiCOVID‐19), which aimed at evaluating the impact of the Covid‐19 pandemic on long‐term care facilities in Catalonia (Amblàs‐Novellas et al. 2024). The Ethics and Research Committee of the University of Vic–Central University of Catalonia approved the project (181/2021). Data collection was conducted from December 2021 to June 2022 in 30 NHs in Catalonia (Spain). NHs were selected according to size, ownership (for‐profit, non‐profit, partial for‐profit/non‐profit), and health region (seeking representativeness of the 10 Catalan regions). Subsequently, 25% of the residents were randomly selected in medium (51–100 beds) and large (> 100 beds) NHs; all residents were invited to participate in small NHs (≤ 50 beds). Participants, or their legal guardians, were informed about the project, and consent was obtained from those willing to participate; if a selected resident (or his/her legal guardian) declined participation, the next resident randomised was selected and invited to participate. Since multilevel analysis requires complete data for both individual and contextual variables, we excluded 8 NHs with missing information on institutional‐related variables. Therefore, we finally included 22 NHs. For this study, subjects under 65 years and those with missing information on bladder and bowel continence status were also excluded.

### 3.3 Variables and Assessment Procedures

Data collection occurred in person and was facilitated by two qualified personnel, either physiotherapists or nurses, from the project who used tablet‐based online questionnaires. Bladder continence during the previous 5 days to assessment was assessed using Section H of the Resident Assessment Instrument—Minimum Data Set version 3.0 (Hawes et al. 1997; Klusch 2012). Residents were classified as continent, occasionally incontinent, frequently incontinent, always incontinent or unclassificable (due to the use of a catheter or other appliance). For the estimation of the prevalence of UI, UI status was dichotomised into ‘no (continent)’/‘yes (occasionally, frequently or always incontinent or using a catheter or appliance)’. The latter variable did not work for multilevel analysis, considering the low variability of the outcome among the NHs. Therefore, we considered the ordinal dependent variable with the categories ‘continent’, ‘occasionally or frequently incontinent’ and ‘incontinent’ (always incontinent or using a catheter or appliance) for inferential analysis. The item on bowel continence status of the MDS was used to classify FI: no (continent) versus yes (occasionally, frequently or always incontinent or using ostomy or appliance). Double incontinence was defined as the presence of both UI and FI (Abrams et al. 2002).

Information related to continence as well as all individual‐level variables were collected through the proxy respondents (NH staff). The selection of professionals most capable of responding to each variable was based on their specific expertise related to the variables in question (Dellefield 2007, 2008). This selection process was pre‐established and organised during the initial meeting with the NH director.

### 3.4 Individual‐Level Variables

Sociodemographic information included age, sex, duration of residence (months), educational level (illiterate, primary, secondary and higher level), and partnership (married or in a relationship versus without partner or widowed). Chronic conditions included oncological, respiratory, cardiac, neurological, hepatic, digestive, and renal diseases. Comorbidity was defined as having 2 or more co‐existent co‐morbid conditions. Individual diagnosed oncological, cardiovascular, neurological and renal conditions (because their potential relation with UI) were considered separately in this analysis. Delirium (presence of delirium and/or a behavioural disorder requiring antipsychotic drugs), falls (≥ 2 falls or hospitalisation due to a fall), polypharmacy (taking ≥ 5 medications), ulcers (presence of vascular and/or pressure ulcers), and dysphagia (difficulty swallowing when eating or drinking; presence of aspiration, respiratory infections) over the previous 6 months were also considered. Constipation was considered when there were fewer than three bowel movements per week and/or specific measures were required to alleviate it (Amblàs‐Novellas et al. 2024). Daily medications were registered; active substances were given an Anatomical Therapeutic Chemical code. Antipsychotic and antidepressant use was identified with codes N05A and N06A, respectively (WHO Collaborating Centre for Drug Statistics Methodology 2012). Participants' total anticholinergic burden (ACB) was measured by summing the ACB score for each drug assessed with the ACB calculator (which assigns drugs a score of 1 for low, 2 for moderate, or 3 for high ACB). Participants were then grouped into five categories for subsequent analyses: no ACB (0), low ACB (1 or 2), moderate ACB (3 or 4), high ACB (5 or 6), or very high ACB (6 or more) (Joshi et al. 2021).

Symptoms of pain and dyspnea as well as sensorial deficits related to sight and hearing (no/yes) were assessed according to the proxy respondent (Amblàs‐Novellas et al. 2018). Nutritional status was assessed through a ≥ 5% loss of body weight or reports of impairment of appetite in the last 6 months (Aprahamian et al. 2017). Emotional markers included the presence of depressive symptoms (feelings of sadness and apathy within the last month) and insomnia/anxiety (nervousness and sleep difficulties experienced in the last month) (Amblàs‐Novellas et al. 2024). Cognitive state was classified with the Global Deterioration Scale (GDS) from 1 (no cognitive impairment) to 7 (very severe cognitive impairment) (Reisberg et al. 1982). The social dimension was explored through indicators of loneliness and social isolation according to the staff's views (Amblàs‐Novellas et al. 2024).

Assessment of basic activities of daily living was conducted using the modified Barthel Index (each item rated on a Likert‐type 5‐point scale from 0 ‘totally dependent’ to 5 ‘totally independent’), excluding bowel and bladder continence (Prado Villanueva et al. 2011; Shah et al. 1989; Moreno‐Martin et al. 2022). Dependence on self‐care was identified if personal hygiene, dressing, or bathing received ratings below 5 in one or more of these items. Dependence on mobility was indicated by ratings below 5 for walking/wheelchair handling or transferring. Dependence on eating was inferred with ratings below 5 in this domain (Moreno‐Martin et al. 2022, 2024). Limitation on instrumental activities of daily living (IADL) was classified if the individual had any difficulties managing money, using the phone and/or handling medication (as these routine tasks are typically unaffected by cultural or gender disparities) (Amblàs‐Novellas et al. 2018).

### 3.5 Institutional‐Level Variables

This information was gathered from NH directors. The NH size was categorised as small (≤ 50 beds), medium (51–100 beds) and large (> 100 beds); NH ownership was classified as for profit and mixed (for profit with public funding). Resident density was calculated from the total number of residents divided by the total m^2^ of each NH. Data on the number of shared rooms and total rooms were gathered to calculate the ratio of shared rooms to total rooms (percentage). The number of shared toilets in relation to the total toilets was calculated to determine the ratio of shared toilets (percentage). Furthermore, data on staffing, including the number of nurses, physiotherapists, occupational therapists, psychologists, social workers, caregivers, nursing assistants, leisure monitors, physicians and geriatricians, along with their weekly hours, were collected. The cumulative hours were then divided by the total number of residents to derive the staffing ratio. Residents' access to outdoor spaces and whether these areas featured natural elements like gardens, trees and plants were collected; these variables were included because of their potential association with residents' health (Wu et al. 2022; Chi et al. 2020).

### 3.6 Statistical Methods

**FIGURE 1:**
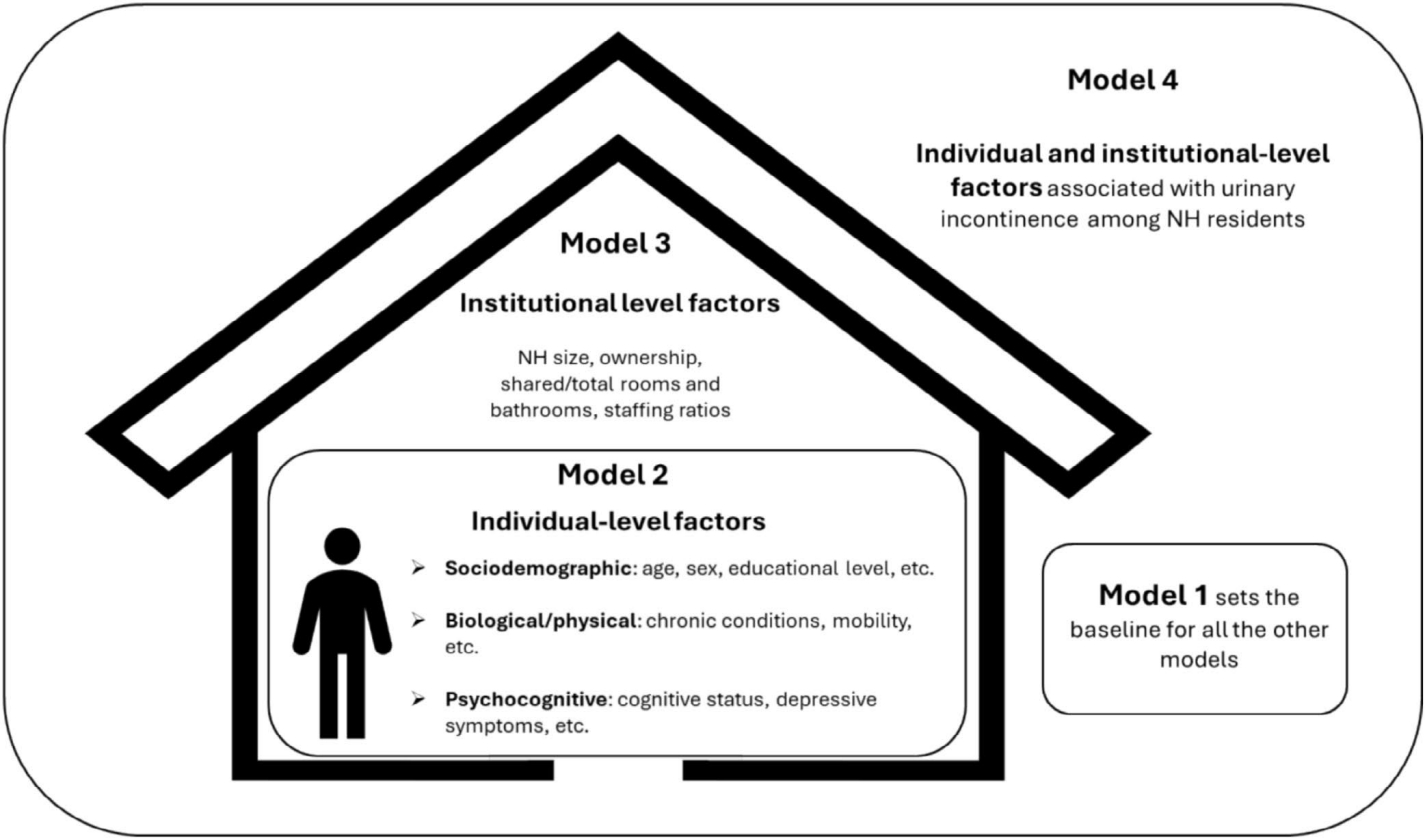
Models considered in the multilevel analysis for UI in NH residents, including individual and institutional‐level factors.

Initially, a descriptive analysis of the data was conducted with summary measures, data tabulation, and graphical analysis. To verify the association of the (ordinal) dependent variable (UI) with all independent variables in the study, a chi‐square trend test was performed. Due to the nature of the outcome and the use of contextual variables related to NHs, a Multilevel Ordinal Logistic Regression model with a random intercept was used as the multivariate analysis strategy, defined according to the results of the likelihood ratio (LR) test. First, an empty model (Model 1) was analysed with only a random intercept. Model 2 included individual‐level variables with a random intercept, and the reduction in the variability of the random effect was examined by comparing it with the previous model. Next, Model 3 included contextual‐level variables with a random intercept. Finally, Model 4 consisted of individual and contextual‐level variables with a random intercept. Figure 1 illustrates the models that were considered in the multilevel analysis.

Statistically significant variables according to the Wald Test (*α* = 0.05), as well as those with theoretical plausibility (i.e., age and sex) were kept in the final model. Interactions at the individual level and between levels 1 and 2 (cross‐level interactions) were also tested (da Silva and Oliveira 2018). To determine the dose–response effect between the independent variables of the study and the outcome variable, a Trend Test (*p* trend) was performed for eligible variables that comprise the final multilevel model (Patino and Ferreira 2016). All analyses were conducted using Stata Software version 15.1 (StataCorp).

## 4 Results

**FIGURE 2:**
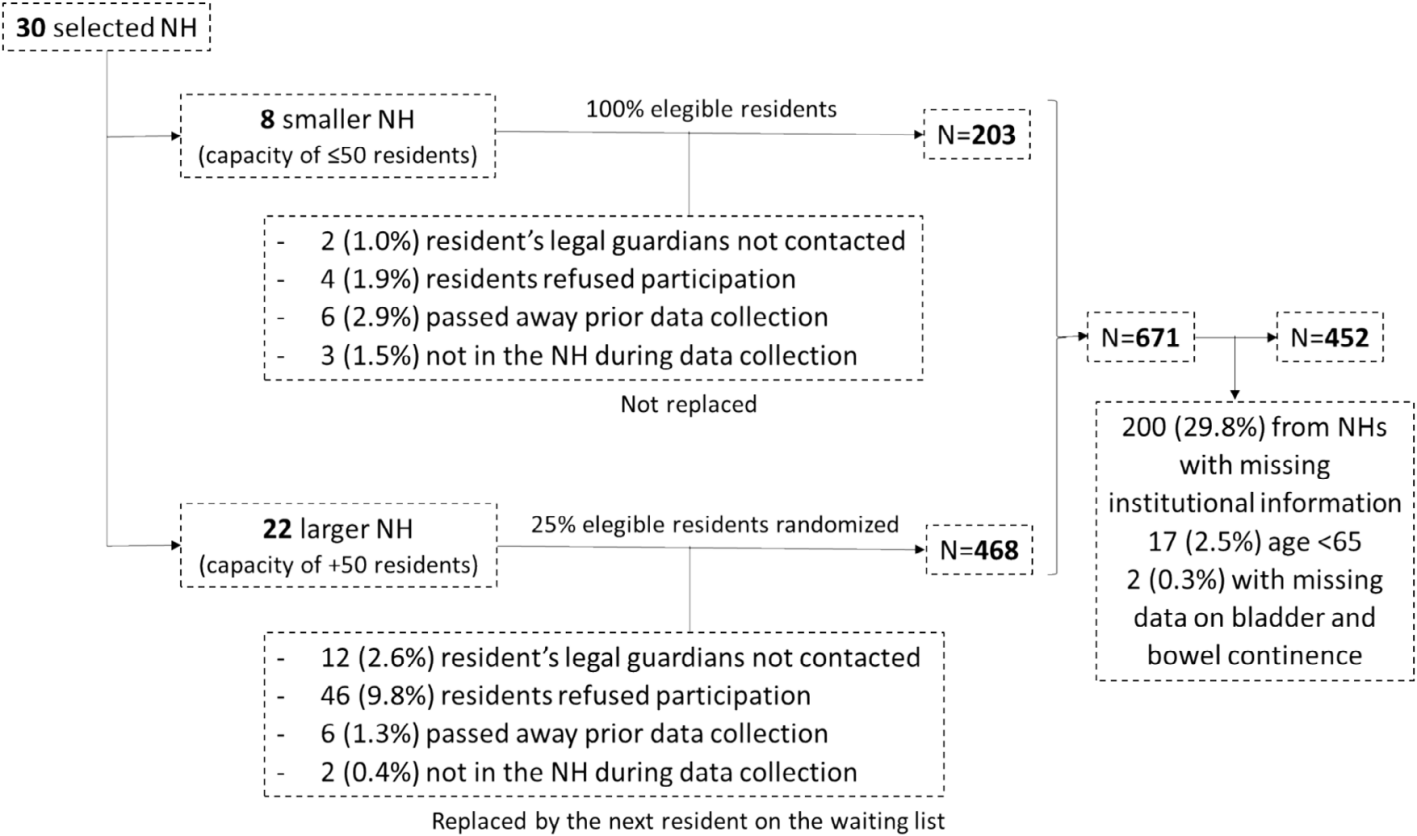
Flowchart of the sampling process.

Figure 2 shows the sampling process. The final sample comprised 452 residents, 75.9% female, with a mean (SD) age of 87.0 (7.7) years and mean (SD) duration of residence of 38.8 (87.5) months. The distribution of UI, FI and double incontinence was 77.5% (95% confidence interval‐CI: 72.4–82.6), 46.1% (95% CI: 40.1–52.2) and 45.7% (95% CI: 39.6–51.7), respectively.

The distribution and unadjusted OR for UI and individual‐level variables among NH residents are presented in Table 1. Statistically significant associations were observed between severe UI and the sociodemographic variables age, sex (women) and education level (lower). Regarding biological/physical factors, mobility limitation, the use of physical restraints, falls, ulcers, dysphagia, constipation and limitations in instrumental activities of daily living were significantly associated with UI. Furthermore, the presence of delirium and behavioural disorders as well as cognitive status (GDS) were the statistically significant psychocognitive variables.

**TABLE 1:** Estimation of OR for UI in NH residents, according to individual‐level variables (*n* = 452).

	UI			OR	95% CI	*p* *
	Continent	Moderate UI	Severe UI			
*Individual variables* (*sociodemographics*)						
Age						
65–85 years	46 (29.9%)	46 (29.9%)	62 (40.2%)	1.00		0.010*
86–91 years	37 (23.0%)	47 (29.2%)	77 (47.8%)	1.41	0.92–2.16	
92–106 years	19 (13.9%)	46 (33.6%)	72 (52.5%)	1.99	1.27–3.12	
Sex						
Woman	67 (19.5%)	112 (32.6%)	165 (47.9%)	1.56	1.02–2.38	0.040*
Men	35 (32.4%)	27 (25.0%)	46 (42.6%)	1.00		
Education (*n* = 26 missing)						
Illiterate	16 (20.0%)	18 (22.5%)	46 (57.5%)	3.04	1.44–6.41	0.014*
Primary	64 (22.1%)	99 (34.3%)	126 (43.6%)	1.88	1.04–3.41	
Secondary‐higher	18 (31.6%)	18 (31.6%)	21 (36.8%)	1.00		
Partnership						
Without partner	86 (22.1%)	125 (32.1%)	178 (45.8%)	0.85	0.49–1.45	0.549
With partner	16 (25.4%)	14 (22.2%)	33 (52.4%)	1.00		
Months of institutionalisation						
0–12	42 (25.4%)	54 (32.7%)	69 (41.9%)	1.00		0.126
13–41	27 (19.4%)	35 (25.2%)	77 (55.4%)	1.61	1.01–2.54	
42 and more	33 (22.3%)	50 (33.8%)	65 (43.9%)	1.18	0.76–1.82	
*Individual variables* (*biological/physical*)						
Comorbidity						
Yes (2+ chronic conditions)	30 (17.0%)	68 (38.4%)	79 (44.6%)	1.29	0.87–1.92	0.198
No	72 (26.3%)	71 (25.9%)	131 (47.8%)	1.00		
Oncological conditions						
Yes	12 (27.3%)	10 (22.7%)	22 (50.0%)	1.18	0.63–2.20	0.602
No	90 (22.1%)	129 (31.6%)	189 (46.3%)	1.00		
Cardiac diseases						
Yes	50 (22.1%)	85 (37.6%)	91 (40.3%)	0.75	0.51–1.09	0.136
No	52 (23.1%)	54 (24.0%)	119 (52.9%)	1.00		
Neurological diseases						
Yes	27 (18.2%)	41 (27.7%)	80 (54.1%)	1.96	1.25–3.07	0.003*
No	75 (24.7%)	98 (32.2%)	131 (43.1%)	1.00		
Renal diseases						
Yes	15 (13.2%)	45 (39.4%)	54 (47.4%)	1.32	0.87–2.01	0.193
No	87 (25.7%)	94 (27.8%)	157 (46.5%)	1.00		
Respiratory diseases						
Yes	14 (18.0%)	26 (33.3%)	38 (48.7%)	1.10	0.68–1.77	0.707
No	88 (23.5%)	113 (30.2%)	173 (46.3%)	1.00		
Digestive diseases						
Yes	8 (25.0%)	14 (43.7%)	10 (31.3%)	0.72	0.37–1.40	0.329
No	94 (22.4%)	125 (29.8%)	201 (47.8%)	1.00		
Polypharmacy						
Yes	87 (21.6%)	127 (31.5%)	189 (46.9%)	1.02	0.55–1.87	0.955
No	15 (30.6%)	12 (24.5%)	22 (44.9%)	1.00		
Antipsychotic use						
Yes	44 (18.4%)	65 (26.8%)	132 (54.8%)	1.99	1.37–2.88	
No	59 (28.0%)	75 (35.6%)	77 (36.5%)	1.00		< 0.001*
Antidepressant use						
Yes	53 (19.5%)	82 (30.7%)	133 (49.8%)	1.40	0.97–2.02	
No	52 (27.9%)	57 (31.2%)	75 (41.0%)	1.00		0.073
Anticholinergic burden						
No‐low	33 (26.0%)	44 (34.6%)	50 (39.4%)	1.00		0.189
Moderate	33 (23.4%)	43 (30.5%)	65 (46.1%)	1.24	0.78–1.97	
High	36 (19.6%)	52 (28.3%)	96 (52.2%)	1.51	0.97–2.36	
Falls						
Yes	6 (11.1%)	19 (35.2%)	29 (53.7%)	1.85	1.03–3.31	0.038*
No	96 (24.1%)	120 (30.2%)	182 (45.7%)	1.00		
Ulcers						
Yes	2 (6.1%)	9 (27.3%)	22 (66.6%)	3.42	1.58–7.39	0.002*
No	100 (23.9%)	130 (31.0%)	189 (45.1%)	1.00		
Dysphagia						
Yes	3 (6.0%)	8 (16.0%)	39 (78.0%)	5.14	2.51–10.55	< 0.001*
No	99 (24.6%)	131 (32.6%)	172 (42.8%)	1.00		
Constipation						
Yes	25 (13.8%)	61 (33.7%)	95 (52.5%)	2.15	1.43–3.23	< 0.001*
No	77 (28.5%)	78 (28.9%)	115 (42.6%)	1.00		
Pain						
Yes	34 (26.4%)	47 (36.4%)	48 (37.2%)	0.67	0.45–1.01	0.055
No	68 (21.0%)	92 (28.5%)	163 (50.5%)	1.00		
Dyspnea						
Yes	8 (20.5%)	14 (35.9%)	17 (43.6%)	0.99	0.53–1.85	0.968
No	94 (22.8%)	125 (30.3%)	194 (46.9%)	1.00		
Hearing problems						
Yes	20 (20.8%)	27 (28.1%)	49 (51.1%)	1.34	0.84–2.13	0.213
No	82 (23.0%)	112 (31.5%)	162 (45.5%)	1.00		
Visual problems						
Yes	16 (27.1%)	22 (37.3%)	21 (35.6%)	0.67	0.38–1.16	0.157
No	86 (21.9%)	117 (29.8%)	190 (48.3%)	1.00		
Weight loss						
Yes	8 (13.3%)	20 (33.3%)	32 (53.4%)	1.41	0.82–2.43	0.216
No	94 (24.0%)	119 (30.4%)	179 (45.6%)	1.00		
Appetite loss						
Yes	4 (9.3%)	14 (32.6%)	25 (58.1%)	1.85	0.97–3.50	0.070
No	98 (24.0%)	125 (30.5%)	186 (45.5%)	1.00		
Physical restraint						
Yes	3 (4.8%)	5 (8.1%)	54 (87.1%)	13.11	5.69–30.18	< 0.001*
No	98 (25.2%)	134 (34.4%)	157 (40.4%)	1.00		
Mobility						
Dependent	58 (15.8%)	112 (30.4%)	198 (53.8%)	12.55	7.05–22.36	< 0.001*
Independent	44 (52.4%)	27 (32.1%)	13 (15.5%)	1.00		
Selfcare						
Dependent	99 (22.3%)	136 (30.6%)	209 (47.1%)			
Independent	3 (100.0%)	0 (0.0%)	0 (0.0%)			
Eating						
Dependent	25 (14.1%)	30 (17.0%)	122 (68.9%)	6.98	4.23–11.52	< 0.001*
Independent	77 (28.0%)	109 (39.6%)	89 (32.4%)	1.00		
IADL						
Dependent on all	43 (13.8%)	85 (27.3%)	183 (58.8%)	5.73	3.73–8.80	< 0.001*
Independent on 1+	59 (42.1%)	53 (37.9%)	28 (20.0%)	1.00		
*Individual variables* (*psychocognitive*)						
Delirium						
Yes	17 (13.2%)	28 (21.7%)	84 (65.1%)	3.30	2.06–5.28	< 0.001*
No	85 (26.3%)	111 (34.4%)	127 (39.3%)	1.00		
Depressive symptoms						
Yes	19 (18.8%)	35 (34.6%)	47 (46.5%)	1.04	0.68–1.61	0.848
No	83 (23.7%)	104 (29.6%)	164 (46.7%)	1.00		
Anxiety symptoms						
Yes	16 (19.8%)	26 (32.1%)	39 (48.1%)	1.11	0.69–1.79	0.655
No	86 (23.2%)	113 (30.5%)	172 (46.3%)	1.00		
Loneliness						
Yes	22 (25.9%)	18 (21.2%)	45 (52.9%)	1.06	0.66–1.71	0.820
No	80 (21.8%)	121 (33.0%)	166 (45.2%)	1.00		
Social isolation						
Yes	18 (25.7%)	21 (30.0%)	31 (44.3%)	0.74	0.45–1.23	0.250
No	84 (22.0%)	118 (30.9%)	180 (47.1%)	1.00		
Cognitive status (GDS)						
GDS 1 (normal)	37 (41.6%)	32 (36.0%)	20 (22.4%)	1.00		< 0.001*
GDS 2 (very mild impairment)	22 (47.8%)	10 (21.7%)	14 (30.5%)	0.99	0.47–2.09	
GDS 3 (mild impairment)	13 (32.5%)	18 (45.0%)	9 (22.5%)	1.35	0.65–2.80	
GDS 4 (moderate impairment)	9 (14.1%)	31 (48.4%)	24 (37.5%)	2.33	1.24–4.38	
GDS 5 (moderate dementia)	8 (15.1%)	20 (37.7%)	25 (47.2%)	3.65	1.83–7.28	
GDS 6 (severe impairment)	10 (10.0%)	22 (22.0%)	68 (68.0%)	8.51	4.52–16.02	
GDS 7 (very severe impairment)	3 (5.0%)	6 (10.0%)	51 (85.0%)	29.29	12.07–71.06	

Abbreviations: CI, confidence interval; OR, odds ratio estimated by the chi‐square trend model.

* *p*, Wald test, statistically significant.

Concerning institutional‐level variables, only the number of physician hours per week was significantly associated with UI in the univariate analysis (Table 2).

**TABLE 2:** Estimation of OR for UI in NH residents, according to institutional‐level variables (*n* = 452).

	UI			OR	95% CI	*p*
	Continent	Moderate UI	Severe UI			
**Institutional variables (structure)**						
Shared/total rooms (%)						
4–67	49 (31.2%)	45 (28.7%)	63 (40.1%)	1.00		0.129
68–91	37 (22.8%)	34 (21.0%)	91 (56.2%)	1.99	1.02–3.89	
92–100	16 (12.0%)	60 (45.1%)	57 (42.9%)	1.47	0.73–2.96	
Shared/total toilets (%)						
0–29	31 (18.8%)	70 (42.4%)	64 (38.8%)	1.00		0.312
30–67	33 (21.7%)	39 (25.7%)	80 (52.6%)	1.64	0.81–3.30	
68–86	38 (28.2%)	30 (22.2%)	67 (49.6%)	1.20	0.57–2.53	
Ownership						
For profit	40 (22.7%)	44 (25.0%)	92 (52.3%)	0.74	0.40–1.37	0.338
Mixed	62 (22.5%)	95 (34.4%)	119 (43.1%)	1.00		
Access to outdoors						
No	8 (53.3%)	2 (18.3%)	5 (33.4%)	0.31	0.07–1.43	0.134
Yes	94 (21.5%)	137 (31.4%)	206 (47.1%)	1.00		
Access to nature						
Yes	28 (15.9%)	56 (31.8%)	92 (52.3%)	1.60	0.88–2.91	0.125
No	66 (25.3%)	81 (31.0%)	114 (43.7%)	1.00		
NH size						
Small (< 50)	39 (23.1%)	42 (24.8%)	88 (52.1%)	1.25	0.61–2.55	0.712
Median (51–99)	24 (22.0%)	42 (38.5%)	43 (39.5%)	0.92	0.44–1.92	
Big (> 100)	39 (22.4%)	55 (31.6%)	80 (46.0%)	1.00		
Density (residents/m^2^)						
0.01–0.02	32 (19.9%)	73 (45.3%)	56 (34.8%)	1.00		0.081
0.03–0.04	26 (17.6%)	34 (23.0%)	88 (59.4%)	2.01	1.05–3.86	
0.05–20.9	44 (30.8%)	32 (22.4%)	67 (46.8%)	1.08	0.56–2.05	
**Institutional variables (NH staff)**						
Total professionals' ratio						
0.00–0.37	37 (24.0%)	60 (39.0%)	57 (37.0%)	0.63	0.29–1.38	
0.38–0.40	22 (19.1%)	41 (35.6%)	52 (45.3%)	0.71	0.31–1.65	
0.41–0.79	31 (23.0%)	33 (24.4%)	71 (52.6%)	1.00		
Physicians' ratio (*n* = 101 missing)						
0.00–0.14	37 (21.4%)	58 (33.5%)	78 (45.1%)	2.72	1.20–6.21	0.034
0.15–0.21	25 (20.7%)	36 (29.7%)	60 (49.6%)	2.93	1.23–7.02	
0.22–0.44	22 (38.6%)	22 (38.6%)	13 (22.8%)	1.00		
Geriatricians' ratio (*n* = 29 missing)						
0.00	59 (19.2%)	101 (32.8%)	148 (48.0%)	1.60	0.80–3.29	0.183
0.01–0.20	34 (29.6%)	37 (32.2%)	44 (38.2%)	1.00		
Nurses' ratio (*n* = 48 missing)						
0.00–0.95	22 (13.2%)	65 (38.9%)	80 (47.9%)	1.43	0.69–2.99	0.106
0.96–1.27	36 (33.3%)	34 (31.5%)	38 (35.2%)	0.64	0.29–1.42	
1.28–5.22	32 (24.8%)	35 (27.1%)	62 (48.1%)	1.00		
Physiotherapists' ratio (*n* = 48 missing)						
0.15–0.41	26 (21.0%)	32 (25.8%)	66 (53.2%)	2.48	1.04–5.89	0.120
0.42–0.59	36 (18.7%)	71 (37.0%)	85 (44.3%)	1.77	0.79–3.97	
0.60–2.31	28 (31.8%)	31 (35.2%)	29 (33.0%)	1.00		
Occupational therapists' ratio (*n* = 48 missing)						
0.00–0.09	49 (22.1%)	72 (32.4%)	101 (45.5%)	0.79	0.33–1.89	0.656
0.10–0.31	21 (21.2%)	43 (43.4%)	35 (35.4%)	0.63	0.23–1.70	
0.32–0.59	20 (24.1%)	19 (22.9%)	44 (53.0%)	1.00		
Psychologists' ratio (*n* = 48 missing)						
0.00–0.20	43 (24.9%)	48 (27.7%)	82 (47.4%)	1.24	0.55–2.78	0.868
0.21–0.32	16 (13.8%)	55 (47.4%)	45 (38.8%)	1.12	0.46–2.74	
0.33–0.89	31 (27.0%)	31 (27.0%)	53 (46.0%)	1.00		
Social workers' ratio (*n* = 48 missing)						
0.07–0.33	20 (15.3%)	44 (33.6%)	67 (51.1%)	1.38	0.66–2.88	0.262
0.34–0.44	23 (23.0%)	48 (48.0%)	29 (29.0%)	0.68	0.31–1.49	
0.45–0.95	47 (27.2%)	42 (24.3%)	84 (43.5%)	1.00		
Nursing assistants' ratio (*n* = 29 missing)						
0.00–0.10	65 (20.9%)	98 (31.5%)	148 (47.6%)	1.33	0.65–2.71	0.433
0.20–29.76	28 (25.0%)	40 (35.7%)	44 (39.3%)	1.00		
Leisure monitors' ratio (*n* = 29 missing)						
0.00	80 (22.9%)	110 (31.5%)	159 (45.6%)	0.96	0.39–2.36	0.927
0.10–0.50	13 (17.6%)	28 (37.8%)	33 (44.6%)	1.00		
Caregivers' ratio (*n* = 48 missing)						
0.00–10.8	30 (21.9%)	60 (43.8%)	47 (34.3%)	0.63	0.27–1.47	0.514
10.9–13.8	40 (23.8%)	43 (25.6%)	85 (50.6%)	0.87	0.38–2.01	
13.9–21.2	20 (20.2%)	31 (31.3%)	48 (48.5%)	1.00		

Abbreviations: CI, confidence interval; OR, odds ratio estimated by the chi‐square trend model; ***p***, Wald test.

Table 3 shows the results of the multilevel analysis. The multilevel modelling, through the initial empty model, provides evidence that, statistically, the variation between NHs is different from zero, according to the LR test (LR 15.41; *p* < 0.001). The results of Model 2 show that statistical significance persisted for most of the variables presented, and the adjustments of OR and their CI compared to the bivariate analysis. The statistical significance persisted in Model 3 (LR 13.31; *p* < 0.001), with a reduction in variance. No institutional variable showed a statistically significant association with UI.

**TABLE 3:** Multilevel analysis between individual and institutional variables for UI in NHs (*n* = 452).

Variables	Model 1	Model 2		Model 3		Model 4	
		OR (95% CI)	*p*	OR (95% CI)	*p*	OR (95% CI)	*p*
**Level 1 (individual factors)**							
Sex							
Woman	—	1.24 (0.77–1.99)	0.374	—	—	1.24 (0.77–1.99)	0.371
Men	—	1.00		—		1.00	
Age	—	1.04 (1.01–1.06)	0.008*	—	—	1.03 (1.01–1.06)	0.010*
Neurological diseases							
Yes	—	2.01 (1.22–3.27)	0.006*	—	—	1.91 (1.16–3.14)	0.011*
No	—	1.00		—		1.00	
Cognitive status (GDS)							
GDS 1 (normal)	—	1.00	< 0.001*	—	—	1.00	< 0.001*
GDS 2 (very mild impairment)	—	1.03 (0.48–2.18)		—		1.01 (0.47–2.13)	
GDS 3 (mild impairment)	—	1.16 (0.55–2.42)		—		1.13 (0.54–2.36)	
GDS 4 (moderate impairment)	—	1.94 (1.01–3.70)		—		1.96 (1.02–3.74)	
GDS 5 (moderate dementia)	—	3.18 (1.58–6.40)		—		3.16 (1.58–6.35)	
GDS 6 (severe impairment)	—	7.15 (3.76–13.62)		—		7.21 (3.78–13.74)	
GDS 7 (very severe impairment)	—	26.01 (10.60–63.78)		—		26.37 (10.74–64.74)	
**Level 2 (institutional factors)**							
Density (residents/m^2^)	—	—	—	0.98 (0.92–1.04)	0.695	0.96 (0.89–1.04)	0.319
Shared/total toilets	—	—	—	0.99 (0.98–1.01)	0.837	1.01 (0.98–1.04)	0.957
Ownership							
For‐profit	—	—	—	1.31 (0.69–2.50)	0.353	1.21 (0.50–5.13)	0.631
Mixed	—	—	—	1.00		1.00	
Fixed effects							
Intercept (95% CI)	0.274 (0.195–0.384)	2.902 (0.629–5.175)		1.261 (1.136–1.786)		2.819 (0.503–5.136)	
Random effects							
Variance (95% CI)	0.342 (0.127–0.920)	0.268 (0.252–1.444)		0.160 (0.109–0.854)		0.247 (0.209–1.322)	
LR test	15.41	26.43		13.31		20.10	
(*x* ^2^. *p*‐value)	(< 0.001)	(< 0.001)		(< 0.001)		(< 0.001)	

Abbreviations: CI, confidence interval; OR, adjusted odds ratio estimated by the multilevel model with random intercept.

* *p*: Wald test; statistically significant.

With the inclusion of all variables (Model 4), the statistical significance was maintained in all variables, except for institutional variables, and adjustments to the OR values were also identified. The variables sex, age and institutional variables were retained in the model due to their theoretical plausibility and the adjustment they provided to the model. For the final model (4), a decline in variance (0.247) compared to Model 1 (0.342) was observed, along with the maintenance of statistical significance (*p* < 0.001 in LR test). UI was significantly associated with neurological conditions, moderate cognitive impairment (GDS 4 level), moderate dementia (GDS 5 level), severe cognitive impairment (GDS 6 level), very severe cognitive impairment (GDS 7 level) and older age. Institutional variables were not significantly associated with UI.

## 5 Discussion

This work had the primary objective of analysing individual and institutional factors associated with UI in older NH residents from Catalonia (Spain). The final model in the multilevel analysis showed individual variables, cognitive impairment, neurological diseases and age to be the significant factors associated with UI. No institutional level variable was associated with UI.

Residents with moderate, severe and very severe cognitive impairment had approximately 2–3, 7 and 26 times (respectively) higher frequency of UI than those with normal cognitive status. Cognitive impairment is one of the associated factors most frequently reported in the literature on UI in NHs (Jerez‐Roig et al. 2024; Offermans et al. 2009). Cognitive deterioration can damage the urination centres in the brain stem and the prefrontal cortex and affect the neural control of the bladder; this mechanism can manifest as urinary urgency and frequency (overactive bladder) which may lead to UI (Tish and Geerling 2020). Cognitively impaired residents may also inconsistently communicate the need to void and are less likely to successfully toilet (Murphy et al. 2021).

Neurological diseases were also statistically significantly associated with UI in the final model. Several studies have found an association of UI with neurological conditions such as stroke or Parkinson's disease among older adults (Cardozo et al. 2023; Jerez‐Roig et al. 2016). Neurological conditions can lead to overactive bladder (linked to urgency UI) as well as mobility impairment and functional decline (Cardozo et al. 2023; Coll‐Planas et al. 2008).

In the present study, it was observed that the mobility variable was not included in the final model due to its predictive interaction with the variables ‘neurological diseases’ and ‘cognitive status’. The relationship between UI and physical disability can be bidirectional. On the one hand, bladder conditions can accelerate the frailty process and lead to a decline in the performance of activities of daily living (Coll‐Planas et al. 2008). Indeed, a recent systematic review reinforces this pathway and identified UI as one of the predictive factors of functional decline among NH residents most commonly reported in the literature (Moreno‐Martin et al. 2022). On the other hand, physical disability impairs mobility, increases sedentary behaviour and may weaken the pelvic floor, leading to UI (Coll‐Planas et al. 2008).

Older versus younger age was statistically significantly associated with UI in our sample, but with a low effect size (OR). Age is one of the strongest risk factors for UI, but in the NH setting (the frailest segment of the population) this factor seems to not play a more important role than cognition and physical disability (Cardozo et al. 2023). This most likely reflects the nature of the medically complex, often frail nature of the NH population. Here, disability associated (functional) UI (cognitive and physical difficulties reaching and using a toilet) is the most frequent type of incontinence.

We performed a multilevel analysis to explore the influence of individual and institutional factors separately and together. According to previous research, the physical environment can affect the quality of life and well‐being of people with dementia, influencing the continence status (Namazi et al. 1991). However, our analysis showed a higher influence of individual factors, and the ratio of physicians to residents was the only institutional‐level variable that reached statistical significance in the bivariate analysis.

A secondary objective of this study was to estimate the prevalence of UI, FI and double incontinence in a representative sample of NH residents in Catalonia. Compared to the international literature, the 77% prevalence of UI found in our study can be considered high (Offermans et al. 2009; Jerez‐Roig et al. 2016; Hoedl et al. 2022). However, it is consistent with the 76% prevalence found in a previous study in 5 NHs of Central Catalonia prior to the covid‐19 pandemic (Jerez‐Roig et al. 2024).

The distribution of FI and DI in our sample, 42% and 57%, was similar to that previously reported in the literature (Blekken et al. 2016; Jerez‐Roig et al. 2015). Approximately 40%–60% of NH admissions with total or partial bowel/bladder control develop dual incontinence in a 1–2‐year period, reflecting a progression in functional disability in this vulnerable population, and for many prevalent UI is later complicated by the development of FI (Bliss et al. 2018). This progression is more rapid for those living with dementia than for those without a dementia diagnosis (Wagg et al. 2016). Deterioration in mobility appears to be a powerful predictor of UI (Jachan et al. 2019).

### 5.1 Strengths and Limitations of the Study

Strengths of this study include the representativeness of the NH sample and its residents. We applied a comprehensive geriatric assessment which included the most important factors associated with UI identified in the literature. Multilevel analysis facilitates the understanding of UI in NHs as a multimethodological and complex condition.

However, this work has a few limitations. Due to the statistical requirements of multilevel analysis, we needed to exclude almost one third of the initial sample because of missing data in institutional‐related variables. Still, half of these variables presented missing data, but the percentage was lower than 10% for the majority. Organisational factors such as staff knowledge on UI or continence care practices were not assessed. Furthermore, COVID‐19 containment measures prevented the collection of information from residents' and therefore all variables were collected through the proxy respondents (NH staff). Due to the epidemiological nature of the Resicovid‐19 project, some health conditions (e.g., nutrition status, social dimension) were assessed with trigger questions, which may have limited the accuracy of responses.

### 5.2 Implications for Practice

Our study indicates that most NH residents present with some impairment of continence, UI being the most prevalent condition. As frontline professionals, nurses are ideally positioned to conduct person‐centred assessments of continence and to recognise early onset of UI. nurses need to ensure that other healthcare professionals are engaged in the management of underlying conditions contributing to UI (such as delirium, constipation, or medication side effects). Given the multiple contributing factors to this geriatric syndrome, the interprofessional team plays a crucial role in providing optimal care.

Care plans should be tailored to each resident's needs, addressing all modifiable factors considering that dependent older residents with moderate–severe cognitive impairment present a higher risk of UI. Multiprofessional collaboration is essential in enhancing continence care quality. Behavioural interventions, such as scheduled toileting, prompted voiding or bladder training, should be adapted according to cognitive and mobility status to encourage successful toileting and combined with appropriate containment strategies to achieve social continence.

## 6 Conclusion

This study showed approximately three out of four NH residents in Catalonia suffer from UI. Furthermore, FI and double incontinence affect almost half of this population. UI was significantly associated with moderate, severe and very severe cognitive impairment, neurological diseases and older age. The multilevel analysis showed that only individual‐level factors, rather than institutional factors, played a role in explaining bladder continence status. These findings highlight the importance of individual‐level interventions to prevent and manage UI in NHs.

## Ethics Statement

Any data has been lawfully acquired in accordance with The Nagoya Protocol on Access to Genetic Resources and the Fair and Equitable Sharing of Benefits Arising from Their Utilisation to the Convention on Biological Diversity. The Ethics and Research Committee of the University of Vic—Central University of Catalonia approved the project with code 181/2021. Data collection was conducted from December 2021 to June 2022 in 30 NHs in Catalonia (Spain).

## Conflicts of Interest

The authors declare no conflicts of interest.

## Data Availability

The data that support the findings of this study are available on request from the corresponding author. The data are not publicly available due to privacy or ethical restrictions.
